# Engineered Extracellular Vesicles Loaded With miR-124 Attenuate Cocaine-Mediated Activation of Microglia

**DOI:** 10.3389/fcell.2020.00573

**Published:** 2020-07-30

**Authors:** Ernest T. Chivero, Ke Liao, Fang Niu, Ashutosh Tripathi, Changhai Tian, Shilpa Buch, Guoku Hu

**Affiliations:** Department of Pharmacology and Experimental Neuroscience, University of Nebraska Medical Center, Omaha, NE, United States

**Keywords:** extracellular vesicles, miR-124, cocaine, microglia, neuroinflammation

## Abstract

MicroRNA-124 (miR-124), a brain-enriched microRNA, is known to regulate microglial quiescence. Psychostimulants such as cocaine have been shown to activate microglia by downregulating miR-124, leading, in turn, to neuroinflammation. We thus rationalized that restoring the levels of miR-124 could function as a potential therapeutic approach for cocaine-mediated neuroinflammation. Delivering miRNA based drugs in the brain that are effective and less invasive, however, remains a major challenge in the field. Herein we engineered extracellular vesicles (EVs) and loaded them with miR-124 for delivery in the brain. Approach involved co-transfection of mouse dendritic cells with Dicer siRNA and RVG-Lamp2b plasmid to deplete endogenous miRNAs and for targeting the CNS, respectively. Mouse primary microglia (mPm) were treated with purified engineered EVs loaded with either Cy5-miR-124 or Cy5-scrambled miRNA oligos in the presence or absence of cocaine followed by assessing EV uptake and microglial activation. *In vivo* studies involved pretreating mice intranasally with engineered EVs followed by cocaine injection (20 mg/kg, i.p.). mPm exposed to EV-miR-124 exhibited reduced expression of miR-124 targets – TLR4 and STAT3 as well as ERK-1/2 and Iba1. In cocaine administered mice, EV-Cy5-miR-124 delivered intranasally were detected in the CNS and significantly reduced the expression of inflammatory markers TLR4, MYD88, STAT3 and NF-kB p65 while also downregulating the microglial activation marker, Iba1. Collectively, these findings suggest that engineered EVs can deliver miR-124 into the CNS, thereby alleviating cocaine-mediated microglial activation. Manipulating EV miRNAs can thus be envisioned as an efficient means for delivery of RNA-based therapeutics to target organs.

## Introduction

Cocaine is a highly potent and addictive brain stimulant that is associated with increased immune reactivity and inflammation in humans (Fox et al., 2012; Ersche et al., 2014; Levandowski et al., 2016; Moreira et al., 2016; Pianca et al., 2017) and neuroinflammation in animal models (Guo et al., 2015; Periyasamy et al., 2017). Worldwide, nearly 22.5 million people are affected by cocaine use disorder (CUD), contributing to increased global health care costs and associated social and economic impact (Pomara et al., 2012). Accumulating evidence suggests a close link between drug abuse and neuroinflammation (Crews et al., 2011; Clark et al., 2013). In particular, cocaine has been shown to activate microglia both in *in vitro* and *in vivo* model systems by mechanisms involving dysregulation of microRNAs (miRNAs) and their target genes (Guo et al., 2016; Periyasamy et al., 2017). A recent study has shown that exposure of microglia to cocaine downregulated the expression of miR-124 with concomitant upregulation of its targets such as TLR4, MYD88 and STAT3 (Periyasamy et al., 2017).

MiR-124 is a brain-enriched miRNA that plays key roles in neurogenesis, synaptic strength and transmission, and glia-neuronal interactions essential for the maintenance of brain homeostasis (Sun et al., 2015; Han et al., 2019). Additionally, miR-124 is highly expressed in microglia and is critical for maintaining microglia in a quiescent state (Ponomarev et al., 2011; Veremeyko et al., 2013; Svahn et al., 2016). Notably, altered expression of miR-124 has been associated with multiple neuroinflammatory disorders such as Parkinson’s (Kanagaraj et al., 2014; Wang et al., 2016; Dong et al., 2018; Yao et al., 2018, 2019), Alzheimer’s Diseases (Lukiw, 2007; Smith et al., 2011; Fang et al., 2012; Zhou et al., 2018), and Huntington’s disease (Johnson and Buckley, 2009). Here we sought to investigate whether restoration of miR-124 in the CNS via intranasal administration of miR-124-enriched EVs could attenuate cocaine-induced neuroinflammation in mice.

Gene therapy is becoming a promising tool for the treatment of human diseases that cannot be cured by conventional therapies. RNA-based approaches such as miRNAs, siRNA, and antisense RNA that are potent sequence-selective inhibitors or activators of transcription are rapidly being developed as therapeutics. EVs that can efficiently shuttle or deliver small molecules between cells have been considered as promising therapeutic delivery conduits for treatment of various diseases, including neurological disorders. Several studies have reported and reviewed the potential of EVs in the delivery of miRNAs for the treatment of various diseases (Zhuang et al., 2011; Vader et al., 2016; Rufino-Ramos et al., 2017; Osorio-Querejeta et al., 2018, 2020; Long et al., 2020). EVs have also been successfully utilized for *in vivo* delivery of siRNAs to specific cell types and tissues in mice (Van Den Boorn et al., 2011; Hu et al., 2018; Liao et al., 2019; Zhao et al., 2020). In the current study, we attempted to deliver exogenous miR-124 to *in vitro* microglia, and to *in vivo* brain in mice, respectively, via engenered EVs released by donor cells depleted of endogenous miRNAs by knockdown of dicer. Intranasal administration of EVs is a potential non-invasive method for rapid delivery of EV-encapsulated drug(s) into the brain via cell uptakes including microglia and other resident CNS immune cells (Visweswaraiah et al., 2002; Lakhal and Wood, 2011; Zhuang et al., 2011; Grassin-Delyle et al., 2012). We envisage that manipulating EVs can be utilized as an efficient, non-invasive means to deliver miRNAs to target organs.

The current study was aimed at testing the hypothesis that intranasal administration of engineered EV-miR-124 could efficiently deliver miR-124 to the CNS microglial cells and thus dampen cocaine-mediated activation and ensuing neuroinflammation. Our findings demonstrated that EV-Cy5-miR-124 was taken up by the microglia *in vitro*, resulting in downregulation of the miR-124 targets – TLR4 and STAT3, and also amelioration of cocaine-mediated microglial activation, as evidenced by downregulation of Iba1 and ERK1/2. Consistently, intranasal delivery of Cy5-miR-124 loaded EVs in cocaine-administered mice not only resulted in attenuation of cocaine-mediated upregulation of TLR4, STAT3, NF-kB p65 and MYD88, but also abrogated cocaine-mediated activation of microglia (Iba1) in the brain. Taken together, our findings suggest that intranasal delivery of engineered EV-miR-124 to the CNS could alleviate cocaine-mediated microglial activation. Manipulating the EV cargo with RNA based therapeutics can thus be envisioned as an efficient, non-invasive means for specific delivery of miRNAs to target organs such as the brain.

## Materials and Methods

### Reagents

Antibodies and reagents used in this work were purchased from the indicated sources: Lamp2 (NB300-591; Novus, Centennial, CO, United States); CD63 (ab216130; Abcam, Cambridge, MA, United States), TLR4 (NB100-56566 Novus, Centennial, CO, United States), Iba1 (Novus NB1001028 or Wako; 19-19741), STAT3 (4904S, Cell Signaling, MA, United States), ERK1/2 (9107S; Cell Signaling, MA, United States), goat anti-mouse-HRP (Santa Cruz Biotechnology; sc-2005), and goat anti-rabbit-HRP (Santa Cruz Biotechnology; sc-2004); NFkB p65 (16502, Abcam, Cambridge, MA, United States); MYD88 (ab2064; Abcam, Cambridge, MA, United States); β-Actin (A1978; Sigma-Aldrich, St. Louis, MO, United States). Cocaine hydrochloride (C5776) was purchased from Sigma-Aldrich, St. Louis, MO, United States. The RVG-Lamp2b plasmid was a gift from Drs. Seow Yiqi and Matthew Wood at the University of Oxford, Oxford, United Kingdom (Alvarez-Erviti et al., 2011).

### Animals

All animal procedures were performed according to the protocols approved by the Institutional Animal Care and Use Committee of the University of Nebraska Medical Centre and the National Institutes of Health. C57BL/6N mice were purchased from Charles River Laboratories (Wilmington, MA, United States) and housed under standard vivarium conditions. Food and water were available *ad libitum* (Chivero et al., 2017). C57BL/6 wild type mice were divided into two groups; receiving either cocaine or saline injections (20 mg/kg, i.p.) for seven consecutive days. Three days prior to sacrifice, mice were intranasally administered dendritic cell-derived EVs loaded with either Cy5-miR-124 or Cy5-scrambled miRNAs or left unloaded, followed by cocaine i.p. injections 1-h post-EV delivery. Mice were sacrificed on the seventh day followed by removal of brain tissues for analysis for protein/RNA analysis and immunohistochemistry.

### Isolation of Mouse Primary Microglia

Mouse primary microglial cells were isolated from C57BL/6N newborn mice pup brains as described previously (Liao et al., 2016; Chivero et al., 2017). Briefly, brain cortices were dissociated and digested in Hank’s buffered salt solution (14025076, Invitrogen, Carlsbad, CA, United States) supplemented with 0.25% trypsin (25300-054, Invitrogen, Carlsbad, CA, United States). Mixed glial cultures were prepared by resuspending cells in culture medium (DMEM supplemented with 10% heat-inactivated fetal bovine serum (FBS) with 100 U/ml penicillin, and 0.1 mg/ml streptomycin) as previously described (Guo et al., 2015). Cells were transferred to T-75 cell culture flasks (10 × 10^6^ cells/flask) and incubated at 37°C and 5% CO_2_ and half of the cell medium was replaced every 3–5 days. At the first medium change, macrophage colony-stimulating factor (GF026, Millipore, St. Louis, MO, United States) was added (0.25 ng/ml) to promote microglial proliferation. Confluent mixed glial cultures (7–10 days) were shaken at 37°C, 220 × *g* for 2 h to promote microglia detachment. Cell medium containing released microglia cells was aspirated, centrifuged at 1000 × *g* for 5 min and collected microglia subsequently plated on cell culture plates for all ensuing experiments. The purity of isolated microglia was confirmed by immunohistochemical staining for Iba1 and was routinely found to be >95% pure (Guo et al., 2015; Chivero et al., 2017).

### DC 2.4 Mouse Dendritic Cells

The DC2.4 mouse dendritic cell line was purchased from Millipore (# SCC142). These cells were routinely maintained at 37°C in 5% CO_2_ in RPMI supplemented with 10 % heat-inactivated fetal calf serum, 1 X L-Glutamine (TMS-002-C, EMD Millipore, MA, United States), 1X non-essential amino acids (TMS-001-C; EMD Millipore, MA, United States), 1X HEPES Buffer Solution (TMS-003-C; EMD, Millipore, MA, United States), 0.0054X β-Mercaptoethanol (ES-007-E; EMD Millipore, MA, United States), 100 IU/ml Penicillin and 100 μg/ml streptomycin.

### Isolation and Quantification of EVs

Extracellular vesicles were prepared from the supernatant fluids of DC2.4 dendritic cells by differential centrifugation as previously described (Hu et al., 2018). In brief, cell culture supernatants were harvested, centrifuged at 1000 × *g* for 10 min to remove dead cells followed by a further spin at 10,000 × *g* for 30 min and subsequent filtration through a 0.22 μm filter to remove any remaining cell debris. Next, the EVs were pelleted by ultracentrifugation (Beckman Ti70 rotor; Beckman Coulter, Brea, CA, United States) at 100,000 × *g* for 70 min. CD63 and Lamp2 were detected by western blot as exosome markers. Purified EV nanoparticle tracking analysis (NTA) was performed using ZetaView Nanoparticle Tracking Analyser (Particle Metrix, Germany) as previously reported (Dagur et al., 2020). Briefly, the instrument was calibrated using the 100 nm polystyrene nano-standard particles followed by loading of 1 mL of each sample (diluted in 0.20 μm filtered PBS). Particle tracking, concentration and video acquisition was carried out with camera sensitivity set to 85, a shutter speed of 100 and a frame rate of 30 according to the manufacturer’s software instructions.

### Small Interfering RNA (siRNA) Transfection

Plasmid and siRNA transfections were performed using Lipofectamine 2000 (11668027; Life Technologies, CA, United States) according to the manufacturer’s instructions. In brief, cells were transfected with plasmid (500 ng) or targeted siRNA (20 pM), mixed with 6 μl of Lipofectamine 2000 diluted in 150 μl Opti-MEM Reduced Serum Medium (31985062; Life Technologies, CA, United States). The resulting siRNA-lipid complexes were added to the cells, incubated for 6 h, and the medium changed into fresh DMEM. Next, the medium was changed to 10% FBS-containing medium for 20 h incubation. The transfected cells were then ready for use in experiments. Sequences of mouse Dicer1 siRNA oligonucleotides used in this study were: mouse Dicer1-si S1, 5′-GrUrGrUrCrArUrC rUrUrGrCrGrArUrUrCrUrArUrUr-3′; mouse Dicer1-si AS1, 5′-UrArGrArArUrCrGrCrArArGrArUrGrArCrArCr UrUr-3′; mouse Dicer1-siS1, 5′-CrCrArArCrUrArCrCrUrCrArUrArUr CrCrCrArUrUr-3′; and mouse Dicer1-si AS2, 5′-UrGrGr GrArUrArU rGrArGrGrUrArGrUrUrGrGrUrUr-3′.

### miRNA Transfection of EVs

Extracellular vesicles were transfected with miRNA using ExoFect Exosome Transfection Reagent (SBI; System Biosciences, Palo Alto, CA, United States) according to the manufacturer’s instructions. Mouse miR-124 oligonucleotides used in this study were synthesized at IDT (Iowa City, IA, United States). Sequences of mouse miR-124 oligonucleotides used in this study were: Cy5-miR-124 – 5′cy5-uaaggcacgcggugaaugcc-cy5-3′; Cy5-scrambled RNA -5′cy5-gaucgaaccuagacuaguggu-cy5-3′, that does not recognize any sequences in mouse transcriptomes. EVs were dissolved in sterile PBS for exposure to microglia or intranasal administration in mice.

### Intranasal Delivery of EVs

C57BL/6N 8 week-old male mice were anesthetized with low dose isoflurane and placed in a supine position in an anesthesia chamber. Delivery of EV-Cy5-miR-124, EV-Cy5-scrambled-miRNA, or control EVs (20 μg/100 μL) via droplets was administered intranasally using a pipette every 2–3 min into alternate sides of the nasal cavity, for a total of 10 min as previously described (Hu et al., 2018). Biodistribution of EVs in the brain, liver and spleen tissues was determined using a Xenogen IVIS 200 imager.

### Western Blotting

Mouse brain tissue homogenates or microglial cells exposed to EVs were lysed with RIPA buffer supplemented with a protease inhibitor cocktail (78430; Thermo Fisher Scientific, CA, United States) followed by ultrasonication for 15 s, at 80% amplitude. Western blotting was performed as previously described (Chivero et al., 2017, 2019). Densitometric analyses were performed using NIH ImageJ software (ImageJ v1.44, NIH) as previously described (Chivero et al., 2019). Protein amounts for bands of interest were normalized to β-actin.

### Immunohistochemistry

Immunofluorescence staining for Iba1 was performed in the whole brain sections of mice intranasally administered dendritic cell-derived EVs loaded with either Cy5-miR-124, Cy5-scrambled miRNA or were left unloaded, followed by 20 mg/kg cocaine injections (*n* = 4). Briefly, four sections from each brain were processed for the immunostaining. The brain sections were washed three times with PBS, permeabilized with 0.3% Triton X-100 in PBS for 30 min as previously described (Liao et al., 2016). The tissues were then blocked with 10% goat serum in PBS for 2 h followed by overnight co-incubation with Iba1 primary antibody at 4°C. Next day, the slides were washed with PBS three times, followed by incubation with corresponding secondary Alexa Fluor 488 goat anti-rabbit (A-11032; Invitrogen, Carlsbad, CA, United States) for 2 h. Finally, the slides were washed with PBS for three times and mounting with ProLong Gold Antifade Reagent with DAPI. Fluorescent images (2–3 images per section) were taken on a Zeiss Observer using a Z1 inverted microscope (Carl Zeiss, Thornwood, NY, United States). The fluorescence intensity, surface area and cell number of Iba-1 + cells were analyzed using the ImageJ software.

### Statistical Analysis

Graphs and statistical analyses were performed using GraphPad software V6.0 (GraphPad Prism Software). Student’s *t*-test was used to compare results between tests and controls. One-way ANOVA was used for multiple comparisons. *P*-values less than 0.05 were considered statistically significant.

## Results

### Characterization of EVs Derived From Dendritic Cells

**FIGURE 1:**
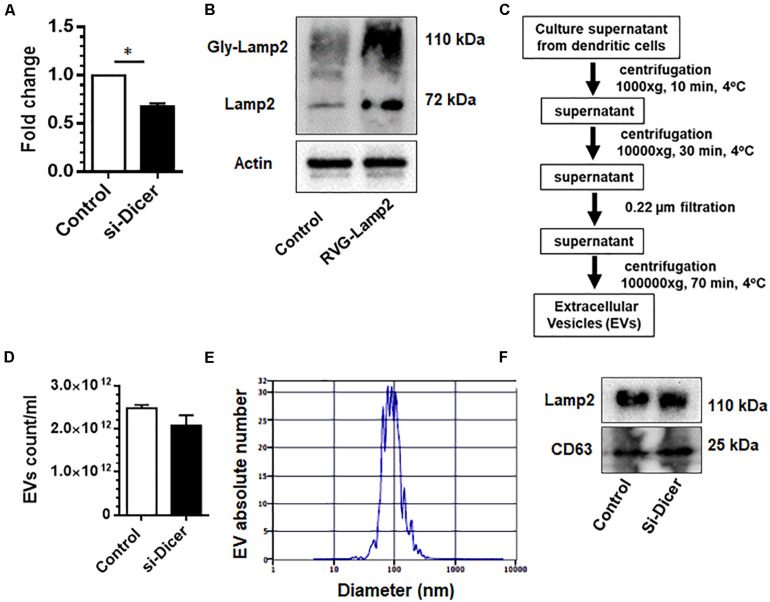
Characterization of dendritic cell-derived EVs: Expression levels of dicer **(A)** and Lamp2b **(B)** in mouse DC2.4 dendritic cell line co-transfected with dicer siRNA and RVG-Lamp2b plasmid. **(C)** Extracellular vesicles (EVs) were isolated from the culture supernatants of engineered DC2.4 mouse dendritic cells by a series of centrifugation. **(D)** Zeta view count of EVs. **(E)** Size distribution of EVs. **(F)** Western blot images for the expression of CD63 and Lamp2 in purified EVs. All experiments were done at least three independent times, and representative figures are shown. Actin served as a loading control for western blots in B. ^∗^*p* < 0.05 vs. control.

Dendritic cells are sentinel antigen-presenting cells of the immune system that produce high levels of EVs. Both dendritic cells as well as EVs derived from dendritic cells are known to possess MHC-I and MHC-II molecules as well as costimulatory molecules, which, in turn, facilitates interaction with immune cells (Pitt et al., 2016). They also possess a variety of surface adhesion membrane proteins, which allow for effective targeting and docking to recipient cells (Pitt et al., 2016). Since dendritic cell-derived EVs can communicate with several cell types, we chose to make EVs from dendritic cells as donor EVs. Herein we first sought to engineer dendric cell-derived EVs by co-transfection of mouse dendritic cells with Dicer siRNA and RVG-Lamp2b plasmid to deplete endogenous miRNAs and for targeting the CNS, respectively. As shown in Figures 1A,B, there was efficient knockdown of dicer and over-expression of Lamp2b in these cells following dicer siRNA and RVG-Lamp2b co-transfection.

We next isolated and characterized EVs from these engineered dendritic cells using a differential ultracentrifugation procedure (Figure 1C). Purified EVs were counted and characterized by their size and by western blot for signature EV markers. As shown in Figure 1D, dicer knockdown by siRNA did not significantly affect the production of EVs as shown by EV counts using Zetaview. EV size ranged from 40 to 150 nm in diameter (Figure 1E). Additionally, levels of selected miRs in these EVs were assessed by qPCR. As shown in Supplementary Figure 1C, levels of selected miR-138 and miR-223 were markedly reduced in EVs isolated from Dicer knockdown cells. Immunoblotting of the EV lysates revealed the presence of exosomal markers CD63 and Lamp2b (Figure 1F).

### Dendritic Cell-Derived EVs Loaded With Cy5-miR-124 Are Taken Up by Microglia and Inhibit Cocaine-Mediated Microglial Activation

**FIGURE 2:**
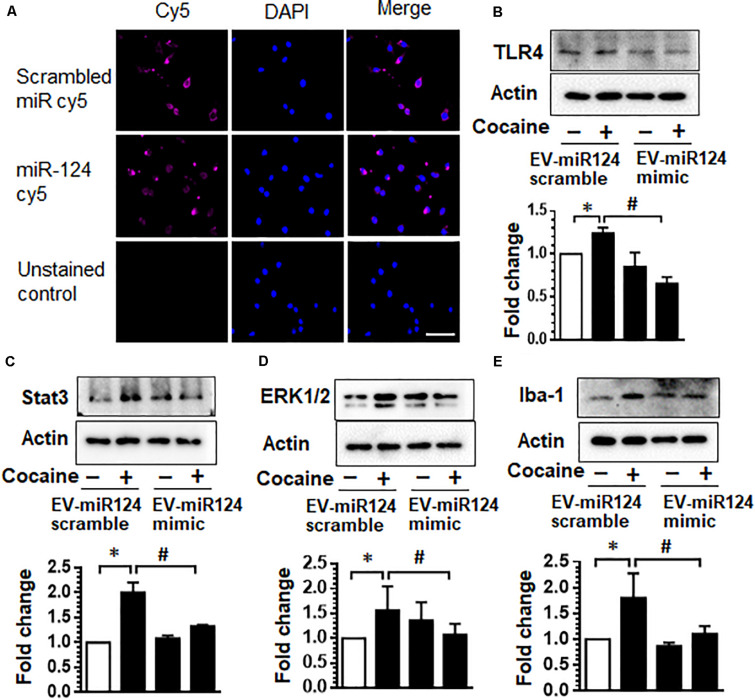
Dendritic cell-derived EVs loaded with Cy5-miR-124 are taken up by microglia and inhibit cocaine-mediated microglial activation. **(A)** Representative images of mouse primary microglia exposed to EVs transfected with Cy5-miR-124 or Cy5-scrambled miRNA (1 h) showing the presence of intracellular Cy5. **(B–E)** Expression of TLR4, STAT3, ERK and Iba-1 in mouse primary microglial cells pretreated with EVs loaded with miR-124 mimic or scrambled miRNA followed by exposure to cocaine (10 μM). All experiments were done at least three independent times, and representative figures are shown. Actin served as a loading control for western blots. Quantification of western blots is shown under each blot. Data are shown as mean ± SEM. ^∗^*p* < 0.05 vs. control, ^#^*p* < 0.05 vs. cocaine group.

We next transfected dendric cell-derived EVs with Cy5-miR-124 mimic oligos and asked whether these EVs could be taken up by microglia, the primary immune cells within the CNS, and inhibit miR-124 target genes that are upregulated upon cocaine exposure. As shown in Figure 2A, mouse primary microglia exposed to EVs transfected with Cy5-miR-124 (1 h) demonstrated a cytoplasmic and perinuclear Cy5 fluorescence suggesting thereby that EVs were taken up by microglial cells (Figure 2A). Since cocaine has been shown to downregulate the expression of miR-124 and consequently upregulate the expression of TLR4 and STAT3, known targets of miR-124 (Periyasamy et al., 2017), we next sought to assess the expression of TLR4 and STAT3 in mouse primary microglial cells exposed to EVs loaded with miR-124 mimic or scrambled miR-124, followed by exposure to cocaine (10 μM). As shown in Figure 2B, exposure of microglia to cocaine in cells exposed to scrambled miRNA-loaded EVs resulted in increased expression of TLR4 compared with control cells. In cells exposed to EVs loaded with miR-124 oligos (EV-miR-124), however, cocaine failed to upregulate the expression of TLR4. As shown in Figure 2C, EVs loaded with miR-124 similarly inhibited the expression of STAT3 in microglia exposed to cocaine compared with cells exposed to cocaine in the presence of scrambled miRNA control.

Having demonstrated that dendritic cell-derived EVs can deliver functional miR-124 to microglia, which, in turn, reduces the expression of key proinflammatory mediators, TLR4 and STAT3, we next sought to investigate the effects of miR-124 on microglial activation. For this mouse primary microglia were exposed to EVs loaded with either miR-124 mimic or scrambled miR-124, followed by exposure of microglia to cocaine and assessed for the expression of Iba1 and ERK1/2 by western blotting. As shown in Figures 2D,E, exposure of microglia to cocaine resulted in significant upregulation of ERK1/2 and Iba1 expression compared with control cells. In the presence of miR-124-EVs, however, cocaine failed to upregulate the expression of both these markers. Collectively, these results imply that miR-124 delivered via EVs blocked cocaine-mediated microglial activation via inhibition of TLR4, STAT3, and ERK1/2 expression.

### Intranasal Delivery of Dendritic Cell-Derived EVs Loaded With Cy5-miR-124 Inhibits Cocaine-Mediated Activation of Microglia *in vivo*

**FIGURE 3:**
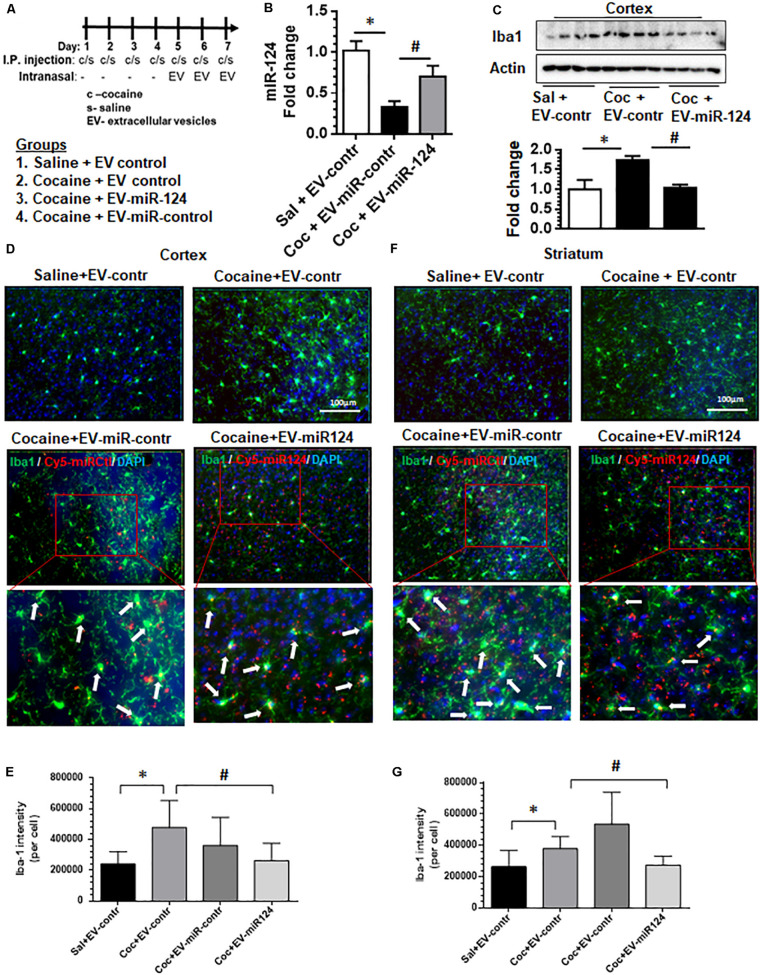
Intranasal delivery of dendritic cell-derived EVs loaded with Cy5-miR-124 inhibits cocaine-mediated upregulation of Iba-1 *in vivo*. Wild-type C57BL/6N mice were administered cocaine (20 mg/kg; i.p.) or saline for seven consecutive days. On the fifth, sixth and seventh days, mice were intranasally administered dendritic cell-derived EVs loaded with Cy5-miR-124 or Cy5-scrambled miRNA or were left unloaded, followed by cocaine injections (i.p.). **(A)** Scheme for intranasal administration of EVs and cocaine injections. **(B)** Expression levels of miR-124 in the cortex. **(C)** Western blot for Iba1 in the cortex. Actin served as a loading control. **(D)** Representative immunofluorescence staining images for Iba1 in mice cortical sections from the four groups of mice i.e., saline + control EVs; cocaine + control EVs; cocaine + EV-Cy5-scrambled miRNA (sham); cocaine + EV-Cy5-miR-124. The blown-out sections show Iba1^+^cy5^+^ microglial cells indicated by arrows. **(E)** Quantification of Iba1 fluorescence intensity normalized to the number of Iba1+ cells in the cortex. **(F)** Representative immunofluorescence staining images for Iba-1 in the striatum from the four groups of mice. **(G)** Quantification of Iba1 fluorescence intensity intensity normalized to the number of Iba1+ cells in the striatum. (*n* = 4/group, ^∗^*p* < 0.05 vs. saline+ EV control, ^#^*p* < 0.05 vs. cocaine+ EV control group, one way ANOVA, Turkey’s multiple comparisons test).

Having demonstrated that dendritic cell-derived EVs can deliver functional miR-124 to microglia and modulate the expression of both proinflammatory and activation mediators *in vitro*, we next sought to validate these observations *in vivo*. Wild-type C57BL/6N mice were administered with either cocaine (20 mg/kg; i.p.) or saline for 7 consecutive days. On the fifth day up to the seventh-day mice were intranasally administered dendritic cell-derived EVs loaded with either Cy5-miR-124 or Cy5-scrambled miRNAs or EVs that were left unloaded, followed by cocaine injections 1 h later (see schematic in Figure 3A). Mice were sacrificed within 4 h after the last injection, perfused with ice-cold PBS, followed by tissue harvesting. The efficacy of EV delivery and biodistribution were monitored using the *in vivo* imaging system (IVIS) and the functional analysis for inflammation and activation pathways in the brain were also performed. As shown in Supplementary Figure 3A, IVIS imaging of organs showed that the Cy5 signal was detectable in the brain and liver at 4 h post-intranasal delivery. We next analyzed for the expression levels of miR-124 and as shown in Figure 3B, levels of miR-124 were reduced in the cortex of mice administered cocaine compared with saline controls. Next, we sought to examine the attenuation of cocaine-mediated upregulation of Iba1 expression in the cortex, by assessing expression of Iba1 by western blotting in mice pretreated with EV-cy5-miR-124 and administered cocaine. As shown in Figure 3C, there was downregulation of Iba1 in the cortex of mice pretreated with EV-Cy5-miR-124 in the presence of cocaine compared with levels of Iba1 in control mice administered cocaine and control EVs. Next, we sought to examine the uptake of labeled EVs by microglia in sections of both the striatum and the cortex by assessing Cy5+ and Iba1+ double immunopositive cells. As shown in Figures 3D,F, Cy5 signal was detected in microglia present in both the cortical and striatal regions (indicated by arrows), thus suggesting the uptake of Cy5-miR-124 EVs by these cells. Interestingly, as expected and as shown in Figure 3D, cocaine administration in the presence of control EVs (intranasal) resulted in upregulated expression of Iba1 in microglia in the cortex compared with that of mice administered with saline and control EVs (intranasal). In mice pretreated with EVs-Cy5-miR-124 intranasally, however, cocaine failed to upregulate the expression of Iba1 (Figure 3D). Quantification of Iba 1 fluorescent intensity normalized to the total number of Iba1^+^ cells in mice brains is shown in Figure 3E. We also analyzed striatal tissue sections and found similar findings as that shown for cortex, specifically that Cy5-miR-124 EVs abrogated cocaine-induced upregulation of Iba1 as evidenced in Figure 3F and quantified in Figure 3G. In addition, we also normalized the surface area of Iba1^+^ cells to the total number of Iba1+ cells and obtained similar results as shown in Supplementary Figures 3C,D.

**FIGURE 4:**
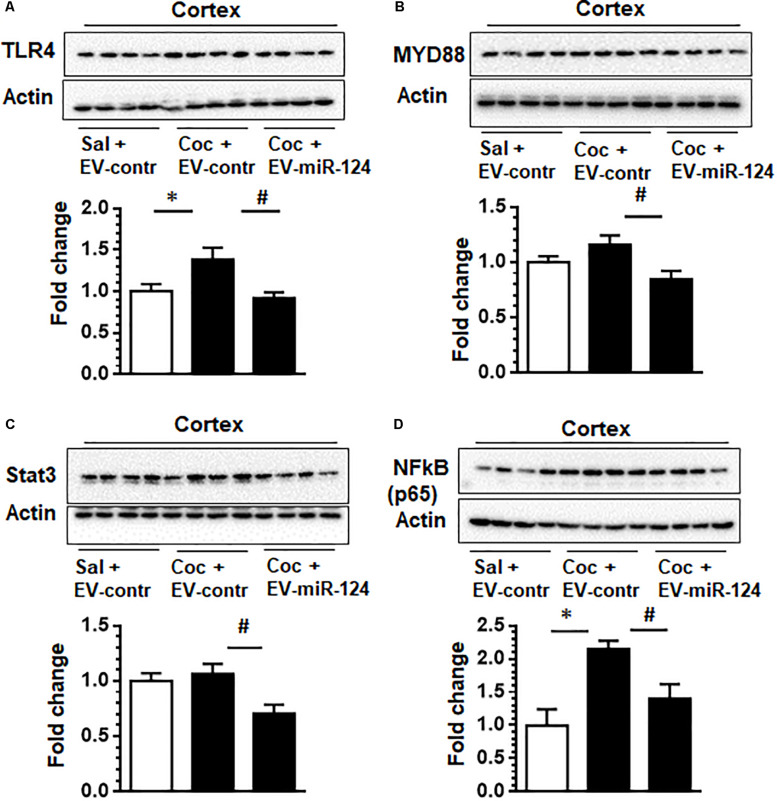
Intranasal delivery of dendritic cell-derived EVs loaded with Cy5-miR-124 inhibits cocaine-mediated upregulation of TLR4, MYD88, STAT3 and NF-kB p65 *in vivo*. Wild-type C57BL/6N, mice were administered either cocaine (20 mg/kg; i.p.) or saline for seven consecutive days. On the fifth, sixth, and seventh days, mice were intranasally administered dendritic cell-derived EVs loaded with Cy5-miR-124 or Cy5-scrambled miRNAs or were left unloaded, followed by cocaine injection. **(A–D)** Western blot images depicting the effect of EV-miR-124 on the expression of TLR4, MYD88, STAT3 and NF-kB p65. Actin served as a loading control. Quantification of western blots is shown under each blot. Data are shown as mean ± SEM (*n* = 4/group, ^∗^*p* < 0.05 vs. saline+ EV control, ^#^*p* < 0.05 vs. cocaine+ EV control group).

Having established the functional effects of Cy5-miR-124 on the expression of Iba1 *in vivo*, we further investigated whether the targets of miR-124 such as TLR4, STAT3, NF-kB p65 and MYD88 could be modulated by exogenous intranasally delivery of EV-Cy5-miR-124 in cocaine administered mice. As shown in Figure 4A and as expected, administration of cocaine resulted in upregulated expression of TLR4 compared with control animals. However, in mice pretreated with EV-Cy5-miR-124, cocaine failed to upregulate the expression of TLR4. Previous studies have shown that miR-124 negatively regulates TLR4-mediated signaling by modulating the expression of MYD88 that is a TLR4 downstream signal (Periyasamy et al., 2017). To confirm whether miR-124 delivered by EVs could also modulate the expression of MYD88, we analyzed for the expression of MYD88 in cortical tissues and found that miR-124 loaded EVs attenuated cocaine-induced upregulation of MYD88 (Figure 4B). Since miR-124 is also known to regulate NF-kB p65 and STAT 3, we also analyzed the expression of these mediators in these mice. The results demonstrated that NF-kB p65 and STAT3 were downregulated in mice administrated with cocaine and EV-Cy5-miR-124 pre-treatment compared with mice administered with cocaine or saline and control EVs, respectively (Figures 4C,D).

## Discussion

Finding an effective and less invasive method for the delivery of microRNA-based drugs into the brain remains a major challenge in the field (Zhuang et al., 2011). In the current study, we loaded EVs produced by dendritic cells with miR-124 for delivery into mouse primary microglia *in vitro* and also for delivery into the brain via the intranasal route in a mouse model of cocaine administration *in vivo*. Dendritic-cell derived EVs were taken up by mouse primary microglia *in vitro* and as well as into the brains of mice following intranasal administration. Increased levels of miR-124 were found to attenuate cocaine-mediated upregulation of TLR4, MYD88, NF-kB p65 and STAT3 – mediators critical for cocaine-mediated neuroinflammation. Additionally, there was also reduced expression of the microglial marker Iba1 in the presence of EV-miR-124. Our results thus suggest that dendritic cell-derived EVs can deliver miR-124 to the CNS, thereby alleviating cocaine-mediated microglial activation.

Dendritic cells are sentinel antigen-presenting cells of the immune system that are known to communicate with neighboring cells through soluble mediators, cell-to-cell-contact and vesicle exchange (Montecalvo et al., 2012) and via release of a vast number of EVs. In addition, dendritic cells and the EVs they produce possess MHC-I and MHC-II molecules as well as costimulatory molecules, which interact with immune cells (Pitt et al., 2016). These cells and their EVs also possess a variety of surface adhesion membrane proteins such as integrins, immunoglobulin family member ICAM-1, and milk fat globule EGF factor 8 (MFG-E8) which facilitates effective targeting and docking to recipient cells (Pitt et al., 2016). Since dendritic cell-derived EVs can communicate with several cell types, herein, we isolated EVs from dendritic cells and loaded them with Cy5-miR-124 for uptake by microglia both *in vitro* and *in vivo*. Here we used EVs derived from the DC2.4 dendritic cell line that has been well characterized and exhibits features of dendritic cells including the expression of dendritic cell-specific markers, cell morphology and has the ability to phagocytose and present exogenous antigens on both MHC class I and class II molecules (Shen et al., 1997). Our findings suggest that microglia can take up these EVs both *in vitro* and *in vivo*. This is particularly relevant since microglia play critical roles in modulating cocaine-induced inflammation (Guo et al., 2015, 2016; Periyasamy et al., 2017). Ability to deliver functional anti-inflammatory miRNAs to these cells could be useful for neuroinflammatory diseases. It was also seen that in addition to microglia other cell types in the brain could also take up EVs. Further studies are warranted to examine the potential side effects of miR-124 in these cells.

Presence and enrichment of miRNAs in EVs have been well-documented (Penfornis et al., 2016; Bayraktar et al., 2017). Endogenous miRNAs in EVs have been shown to affect the cellular functions of recipient cells via various mechanisms (Bayraktar et al., 2017). Furthermore, knockdown of dicer in donor cells has been shown to deplete the presence of miRNAs in the EVs isolated from these dicer knock out cells (Squadrito et al., 2014). In the current study, we attempted to limit the off-target effects of miRNAs in EVs by knocking down dicer using the siRNA approach. We do acknowledge that besides miRs, EVs are also endowed with cargo containing proteins and lipids that could lead to off-target effects that need to be yet characterized. As a means to increase the specific delivery of EVs into the CNS, we followed the approach reported by Alvarez-Erviti et al. (2011) wherein the authors were able to successfully express Lamp2b, an EV membrane protein, fused to an rabies virus glycoprotein (RVG) peptide in dendritic cells, followed by loading the EVs isolated from these cells with exogenous siRNA of GAPDH. RVG peptide is known to target the alpha-7-subunit of the nicotinic acetylcholine receptor (Kim et al., 2010), and facilitates the transcytosis of the EVs across the blood-brain barrier, with specific targeting of neural cells in the brain (Loddo et al., 1966; Alvarez-Erviti et al., 2011). It was reported that intravenously injected RVG EVs delivered GAPDH siRNA specifically to the neurons, microglia and oligodendrocytes in the brain, resulting in tissue-specific gene knockdown (Alvarez-Erviti et al., 2011). In agreement with these findings, our data also demonstrated delivery to the brain of Cy5-miR-124 using these Lamp2-RVG tagged EVs.

It has been well-recognized that miR-124 is highly expressed in microglia and is critical for maintaining microglia in a quiescent state (Ponomarev et al., 2011; Veremeyko et al., 2013; Svahn et al., 2016). Cocaine has been shown to downregulate expression of miR-124 in microglia with concomitant upregulation of its targets – KLF4 and TLR4 (Guo et al., 2016; Periyasamy et al., 2017). Interestingly, overexpression of lentivirus miR-124 by stereotactic injection was shown to inhibit microglial activation in cocaine-administered mice (Periyasamy et al., 2017). The current study was aimed at developing a non-invasive method for miR-124 delivery into the brain using brain targeted EVs. Our *in vitro* findings suggested that a physiologically relevant dose of cocaine (10 μM) resulted in significant upregulation of proinflammatory mediators (TLR4, STAT3) and that this effect was attenuated by EV-miR-124. It has been shown that the plasma levels of cocaine in humans who inhale cocaine intranasally ranges between 0.4 and 1.6 μM (Van Dyke et al., 1976) while the plasma cocaine levels in tolerant abusers was found to be 13 μM (Stephens et al., 2004). Further, the levels of cocaine in the postmortem brains of chronic cocaine users with acute intoxications have been found to be greater than 100 μM (Kalasinsky et al., 2000). Cocaine concentrations used in this study are in keeping with the physiological levels observed in humans abusing cocaine. Our *in vivo* findings demonstrated that intranasal delivery of EVs loaded with miR-124 could reach the brain, leading, in turn, to downregulated expression of TLR4 and other miR-124 target genes, ultimately resulting in amelioration of microglial activation in cocaine-administered mice.

In summary, our findings demonstrate that intranasal delivery of CNS target peptide-engineered EVs (from dicer-deficient dendritic cells) loaded with miR-124 have the potential to abrogate cocaine induced-microglial activation *in vivo*. In summary, manipulating EV miRNAs can be envisioned as an efficient means for RNA drug delivery to target organs, such as the brain, for the treatment of neuroinflammatory diseases such as cocaine addiction.

## Data Availability Statement

All datasets presented in this study are included in the article/Supplementary Material.

## Ethics Statement

The animal study was reviewed and approved by the UNMC Institutional Animal Care and Use Committee.

## Author Contributions

EC, FN, AT, KL, and CT performed the experiments and collected, analyzed, and discussed the data. SB, EC, and GH designed the experiments, discussed the data, and drafted and revised the manuscript. All authors have read and approved the final manuscript.

## Conflict of Interest

The authors declare that the research was conducted in the absence of any commercial or financial relationships that could be construed as a potential conflict of interest.
