# Supplementary material for: Engineered Extracellular Vesicles Loaded With miR-124 Attenuate Cocaine-Mediated Activation of Microglia
Source: Front Cell Dev Biol. 2020 Jul 30;8:573. doi: 10.3389/fcell.2020.00573 (PMC7409518; doi:10.3389/fcell.2020.00573)
Supplement: Supplementary file 1 [file Data_Sheet_1.pdf]

Supplementary Figure 1

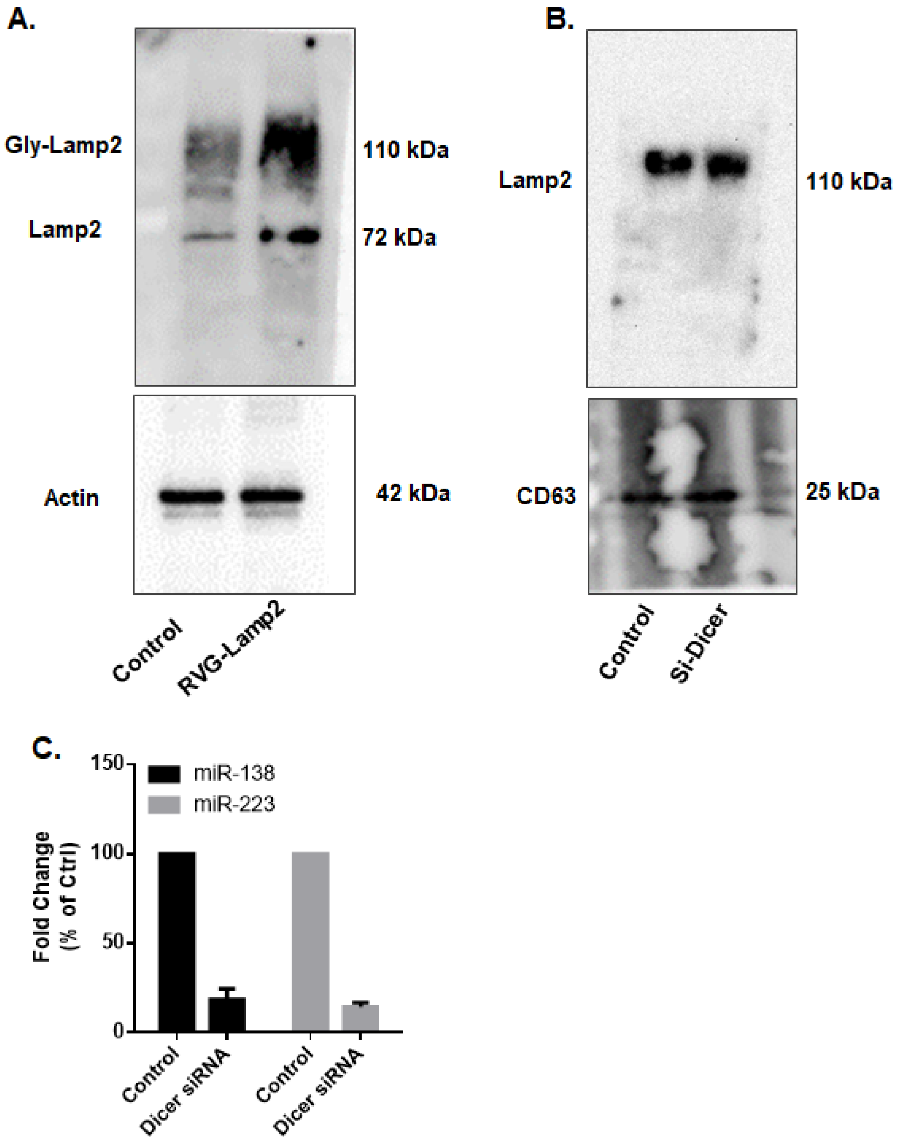

Supplementary Figure 1. Full western blot images for the expression of **(A)** Lamp2 and actin in DC 2.4 dendritic cells, **(B)** Lamp2 and CD63 in purified EVs. **(C)** Cells were transfected with either siRNA-control or Dicer-siRNA for 24h followed by EV isolation and detection of indicated miRs by qPCR. Data show fold change with Ct values normalized to U6.

Supplementary Figure 2

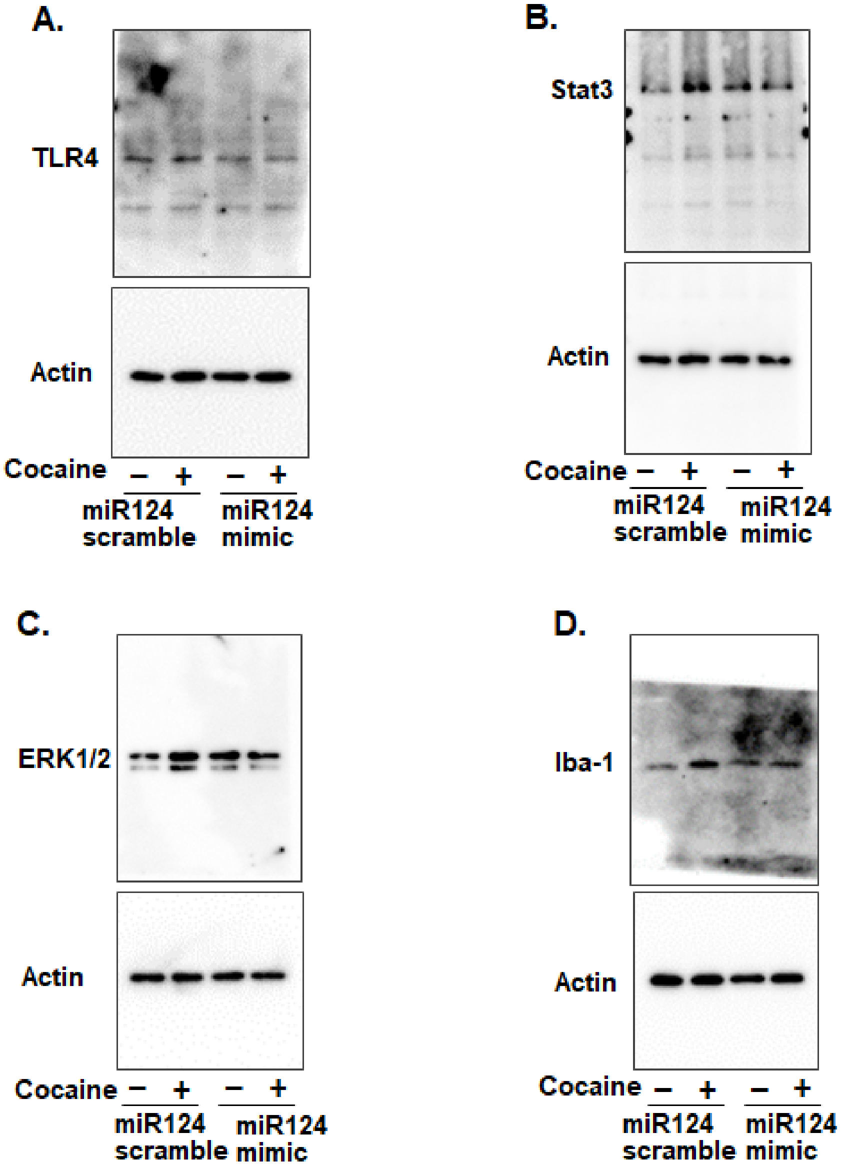

Supplementary Figure 2. Full western blot images for the expression of TLR4 **(A)**, Stat3 **(B)**, ERK **(C)** and Iba-1 **(D)** in mouse primary microglial cells pre-treated with EVs loaded with miR-124 mimic or scrambled miR-124 followed by exposure to cocaine (10 µM).

Supplementary Figure 3

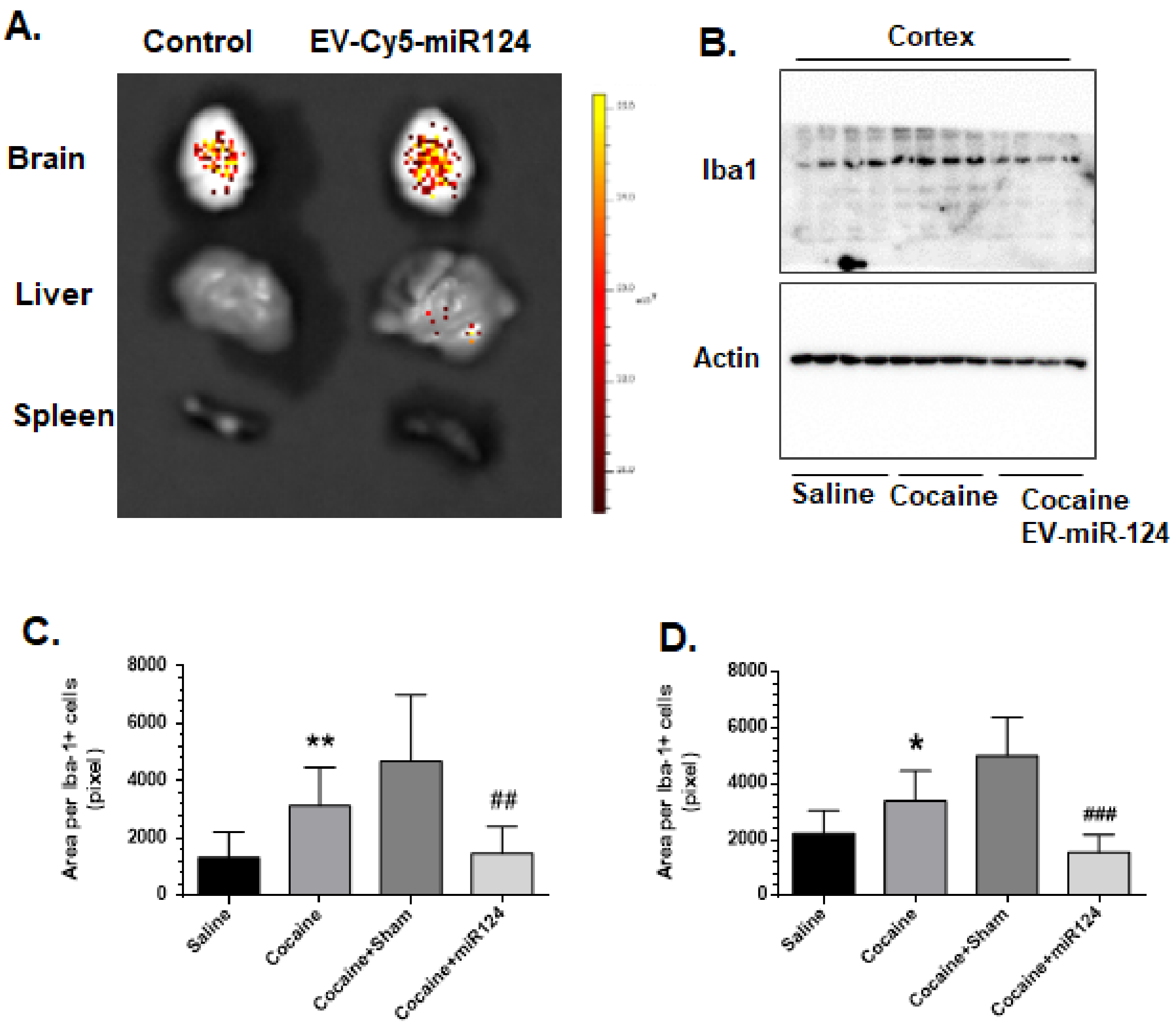

Supplementary Figure 3. IVIS imaging of brain, liver and spleen **(A)**. Full western blot for Iba1 in the cortex **(B)**. Quantification of Iba1 surface area normalized per Iba1 positive cells in the cortex **(C)** and striatum **(D)**. (n = 4/group, * $p < 0.05$ vs. control, # $p < 0.05$ vs. cocaine group).

Supplementary Figure 4

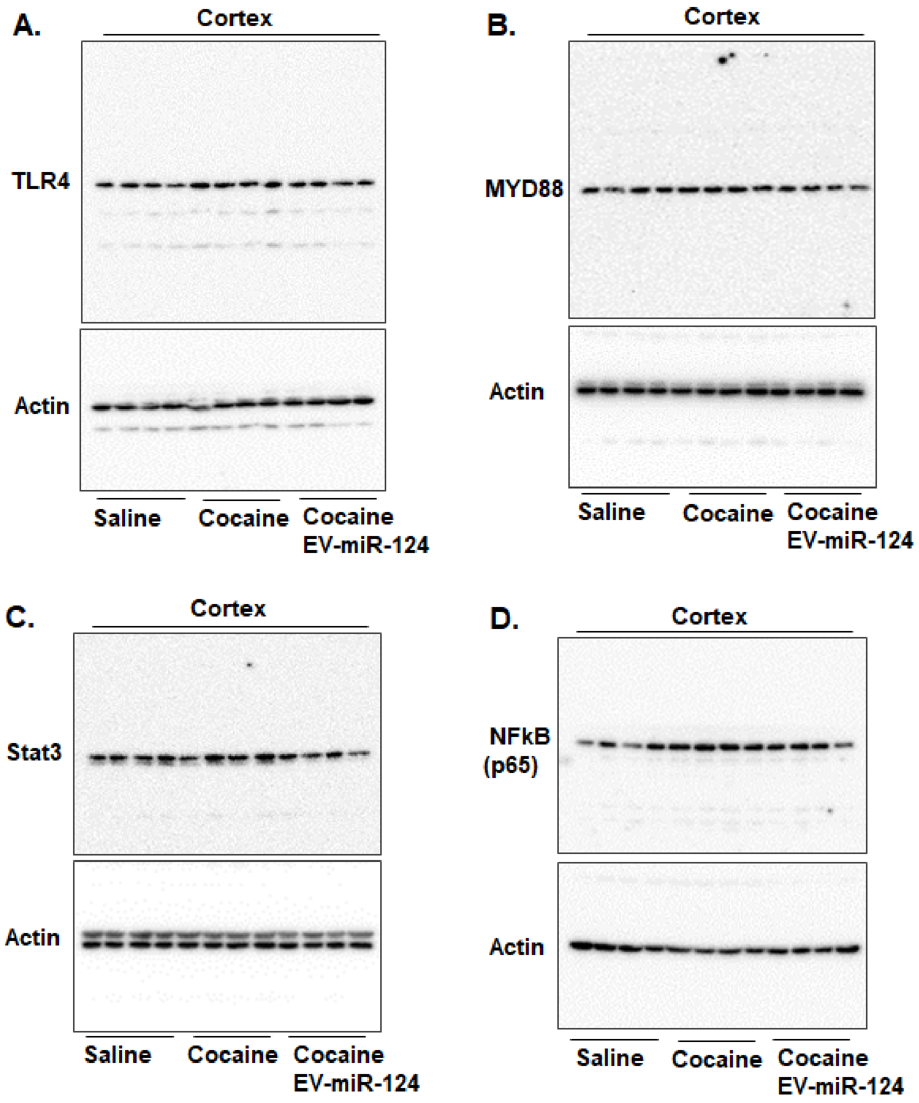

Supplementary Figure 4. Full western blot images showing the effect of EV-miR-124 on the expression of TLR4 **(A)**, MYD88 **(B)**, Stat3 **(C)** and NFkB p65 **(D)**.
